# Structural Features of Carnivorous Plant (*Genlisea*, *Utricularia*) Tubers as Abiotic Stress Resistance Organs

**DOI:** 10.3390/ijms21145143

**Published:** 2020-07-21

**Authors:** Bartosz J. Płachno, Saura R. Silva, Piotr Świątek, Kingsley W. Dixon, Krzystof Lustofin, Guilherme C. Seber, Vitor F. O. Miranda

**Affiliations:** 1 Department of Plant Cytology and Embryology, Institute of Botany, Faculty of Biology, Jagiellonian University in Kraków, Gronostajowa 9 St. 30-387 Cracow, Poland; krzysztof.lustofin@doctoral.uj.edu.pl; 2 Laboratory of Plant Systematics, School of Agricultural and Veterinarian Sciences, São Paulo State University (Unesp), Jaboticabal, CEP 14884-900 SP, Brazil; saura.silva@gmail.com (S.R.S.); gcseber@gmail.com (G.C.S.); vitor.miranda@unesp.br (V.F.O.M.); 3 Faculty of Natural Sciences, Institute of Biology, Biotechnology and Environmental Protection, University of Silesia in Katowice, Jagiellońska 28, 40-032 Katowice, Poland; piotr.swiatek@us.edu.pl; 4 School of Molecular and Life Sciences, Curtin University, Kent Street, Bentley, Perth, WA 6102, Australia; kingsley.dixon@curtin.edu.au

**Keywords:** plant anatomy, abiotic stress, bud-bearing structures, Brazilian *Cerrado*, *campo rupestre*, carnivorous plants, *kwongan*, Lentibulariaceae, starch grains, tubers

## Abstract

Carnivorous plants from the Lentibulariaceae form a variety of standard and novel vegetative organs and survive unfavorable environmental conditions. Within *Genlisea*, only *G. tuberosa*, from the Brazilian *Cerrado*, formed tubers, while *Utricularia menziesii* is the only member of the genus to form seasonally dormant tubers. We aimed to examine and compare the tuber structure of two taxonomically and phylogenetically divergent terrestrial carnivorous plants: *Genlisea tuberosa* and *Utricularia* *menziesii*. Additionally, we analyzed tubers of *U. mannii*. We constructed phylogenetic trees using chloroplast genes *mat*K/*trn*K and *rbc*L and used studied characters for ancestral state reconstruction. All examined species contained mainly starch as histologically observable reserves. The ancestral state reconstruction showed that specialized organs such as turions evolved once and tubers at least 12 times from stolons in Lentibulariaceae. Different from other clades, tubers probably evolved from thick stolons for sect. *Orchidioides* and both structures are primarily water storage structures. In contrast to species from section *Orchidioides*, *G. tuberosa*, *U.* *menziesii* and *U. mannii* form starchy tubers. In *G. tuberosa* and *U. menziesii*, underground tubers provide a perennating bud bank that protects the species in their fire-prone and seasonally desiccating environments.

## 1. Introduction

The Lentibulariaceae are carnivorous plants that produce three types of traps: flypaper (*Pinguicula*), eel (*Genlisea*), and suction (*Utricularia*) (e.g., [1,2,3,4]). *Genlisea* and *Utricularia* exhibit a novel plant architecture (e.g., [5,6,7,8]) linked to their growth in wet habitats. However, some are exposed seasonally to abiotic stresses such as drought, high temperatures or frost. To survive unfavorable ecological conditions, members of the Lentibulariaceae form various vegetative organs such as: hibernaculae, dormant rosettes, subterranean bulb-like rosettes, turions, thick stolons, stem and tubers.

Hibernacula occur in temperate species like *Pinguicula vulgaris*, *P. grandiflora* and *P. alpina*. The hibernaculum consists of an abbreviated stem-bearing scales and leaf primordia. Reserves (starch) are stored mainly in swollen hibernaculum scales (*P. vulgaris* and *P. grandiflora*) or in the stout perennial roots (*P. alpina*) [9]. Dormant rosettes and subterranean bulb-like rosettes occur in the heterophyllous *Pinguicula* species of Mexico and Central America. Leaves in these rosettes are non-carnivorous have a reduced lamina and possess succulent characteristics (e.g., [10]).

In the case of *Genlisea*, the stress resistance organs might be: scapes, thick stems, leaves and tubers. Large, thick and succulent photosynthetic scapes occur in *Genlisea uncinata* and *G. oligophylla* [11,12]. According to Fleischmann [12], *Genlisea metallica* survives seasonal aridity by a perennating short, succulent vertical underground stem. Rivadavia [11] noted that *Genlisea aurea* may form the stem up to approximately 1.5 cm in length by 4 mm in diameter and this thickened structure may also provide drought protection. Thick, succulent leaves occur in *G. uncinata* (Fleischmann, 2012). However, among the members of *Genlisea*, *G. tuberosa* is unique in forming tubers ([11] as found in *G. pygmaea*; [12,13]). According to Rivadavia [11] each plant produces a single tuber. These stolon-derived tubers arise in the early stages of seedling development [12], however there are no data on the anatomy and morphological characters in *Genlisea* tubers.

In *Utricularia,* stress resistance organs include turions, thick stolons and tubers [14]. The ten species of aquatic *Utricularia* from sect. *Utricularia* form turions in response to unfavorable ecological conditions, usually at the beginning of autumn [14,15]. The *Utricularia* turion is an overwintering organ, which is formed by the condensation of short, modified leaves in the shoot apex. In comparison to other tissues, the turion has highly reduced metabolism and contains starch as the primary reserve material and protein (e.g., [14,15,16,17,18,19]). According to Taylor [14], tubers in *Utricularia* occur mostly in the epiphytic and lithophytic species from sect. *Orchidioides*, *Phyllaria*, *Chelidon* and this organ is important for surviving the dry season. Thick stolons also occur in some *Utricularia* species (*U. reniformis*, *U. humboldtii*, *U. nelumbifolia, U. cornigera*) from former sect. *Iperua* (now included in sect. *Orchidioides*) and provide drought resistance [14,20,21]. Thick stolons occur in some species from sect. *Pleiochasia* with the root tubers of *Utricularia menziesii* being unique [14,22]. This terrestrial species survives the dry season by senescing to quiescent over-summering tubers [14,23]. Tubers or tuber-like structures have been recorded in aquatic species *U. benjaminiana*, *U. reflexa*, *U. inflata*, and *U. radiata* [14,20]. However, in these aquatic species sect. *Utricularia* tubers perhaps may function as vegetative propagules similar to the turions. According to Rice [20], there are two types of *Utricularia* tubers: water storage and carbohydrate-rich tubers. Rice [20] outlined that *Utricularia menziesii* possessed carbohydrate-rich tubers, however, he did not provide histochemical evidence and also no literature sources for this information. Pate and Dixon [22] studied mineral and carbohydrate content of *U. menziesii* tubers and highlighted that these act as the primary over-summering phase for the species though no histological analysis was provided. For annual species of *Genlisea* and *Utricularia*, the primary drought tolerance mechanism is the production of a soil seed bank [24]. Besides the drought tolerance, the small seed size associated with large testa cells with a foveolate surface of *Genlisea* and *Utricularia* species permits aggregation of air bubbles and thereby facilitating the floatability and the dispersion through the water channels produced by seasonal rains in the *Cerrado* [25].

**Figure 1:**
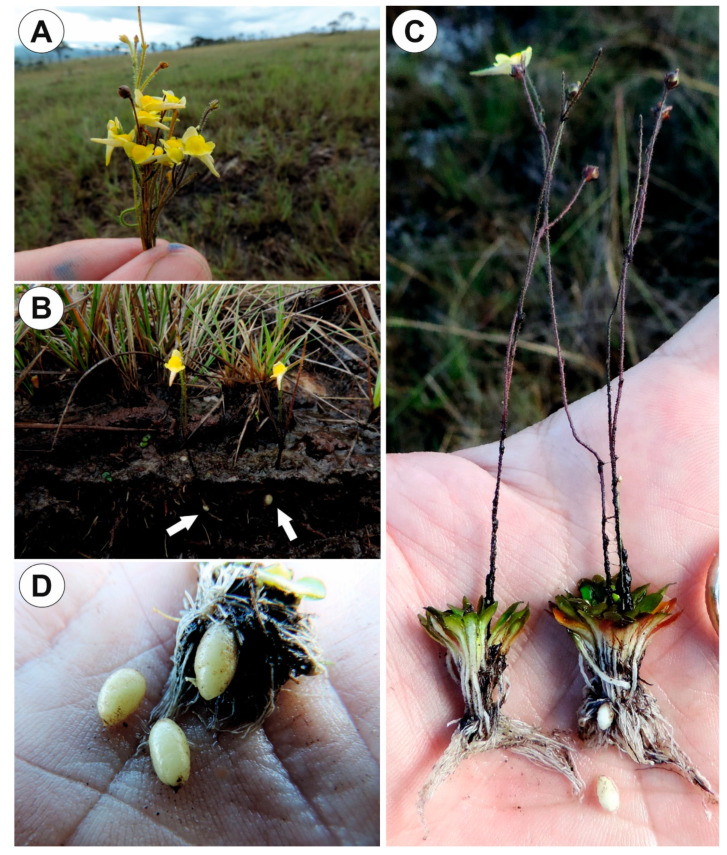
*Genlisea tuberosa* in its natural habitat, the Serra da Canastra, southern Minas Gerais State (south-eastern Brazil), in the rocky fields of the *Cerrado*. (**A**). Inflorescences of *Genlisea tuberosa* in the Serra da Canastra. (**B**). Exposed traps and tubers (arrows). (**C**). Two excavated plants. (**D**). Tubers.

*Genlisea tuberosa* and *Utricularia menziesii* were selected for this study as they occur in special fire-prone environments. Both are exposed to high irradiance and air temperatures during the dry season and aestivate using underground tubers. These species occur in the old climatically-buffered infertile landscapes (OCBILs, [26]); *Genlisea tuberosa* in *campos rupestres* (rupestrian grasslands) (Figure 1A–C) and *Utricularia menziesii* in *kwongkan* of south-western Australia. *U. menziesii* belongs to sect. *Pleiochasia*, within *U.* subgen. *Polypompholyx* [27,28], an early-branching lineage which has unexpected features such as specialization for bird pollination [29]. Therefore, compared tuber structures from the divergent lineages in Lentibulariaceae (*Genlisea* genus and *Utricularia* subgen. *Polypompholyx* sect. *Pleiochasia*) with tuberous structures of species (subgen. *Utricularia* sect. *Orchidioides*, [21]) that are thought to be primarily for storage of water [21,30,31]. Additionally, we chose tubers of *Utricularia mannii* (sect. *Chelidon*) as Taylor [14] noted that this species has many common features (including tuber formation) with species from sect. *Orchidioides* (subgen. *Utricularia*). However, according to the phylogeny based on *trn*K/*mat*K and *trn*L-F DNA sequences [32] *U. mannii* is nested within subgen. *Bivalvaria* (sensu [33]). This species, in contrast to the terrestrial *Genlisea tuberosa* and *Utricularia menziesii*, is an epiphyte found on Mount Cameroon, Cameroon, Africa. Thus, our study aims to examine and compare the tuber structures from the divergent lineages in Lentibulariaceae, represented by *Genlisea tuberosa*, *Utricularia menziesii* and *U. mannii*, to more fully understand the relationship between anatomical features as related to phylogenetic links and perennation mode.

## 2. Results

### 2.1. Genlisea tuberosa Rivadavia, Gonella & A. Fleischm

**Figure 2:**
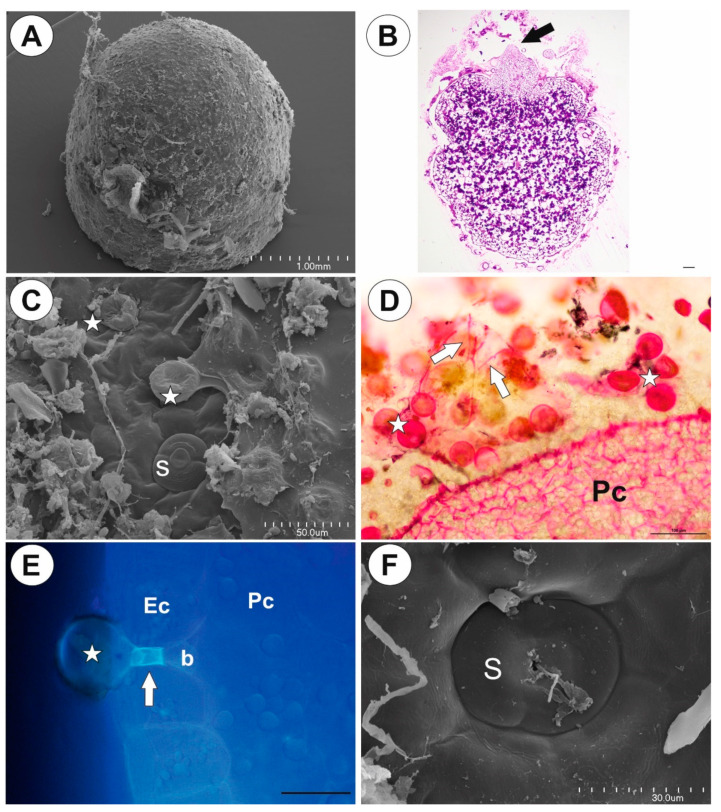
Structure of *Genlisea tuberosa* tubers. (**A**). Scanning electron micrograph (SEM) of a tuber; scale bar = 1 mm. (**B**). Longitudinal section through the tuber, note bud (arrow), PAS (periodic acid-Schiff) reaction; scale bar = 100 µm. (**C**). SEM micromorphology of the tuber surface: glandular trichomes (star), stoma (S); scale bar = 50 µm. (**D**). A tuber section stained with ruthenium red: glandular trichomes (star), fungal hyphae (arrows), parenchyma (Pc); scale bar = 100 µm. (**E**). Structure of epidermal glandular trichome, note fluorescence of the pedestal cell that is heavily impregnated with cutin: basal cell (b), pedestal cell (arrow), head cell (star), epidermal cell (Ec), parenchyma cell (Pc); scale bar = 60 µm. (**F**). SEM, stoma from tuber surface; scale bar = 30 µm.

**Figure 3:**
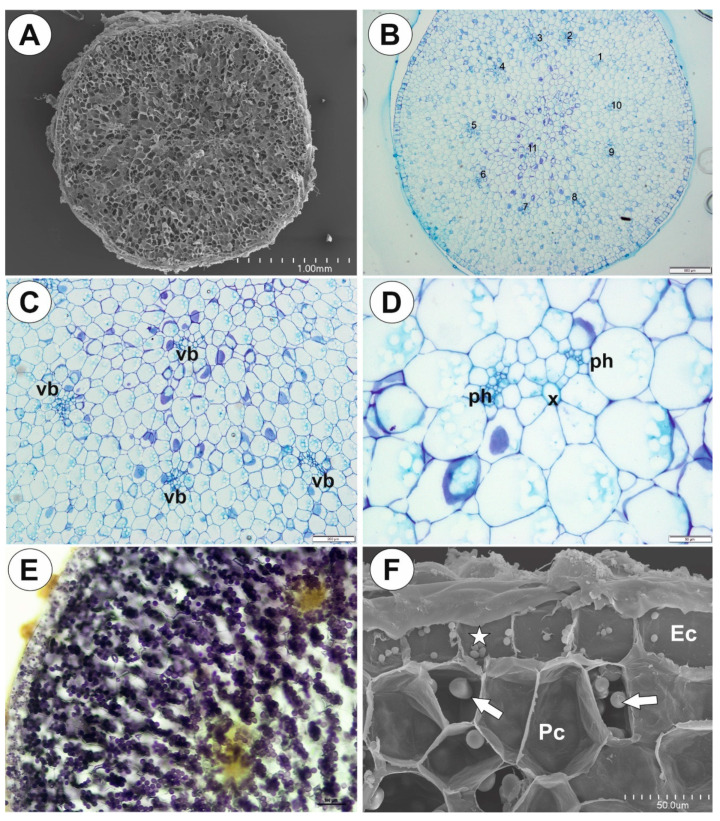
Anatomy and histochemistry of *Genlisea tuberosa* tubers. (**A,B**). General tuber anatomy. Note the numerous vascular bundles (numbers), A. SEM; scale bar = 1mm and 500 µm. (**C,D**). Vascular bundles (vb): phloem (ph), xylem vessel (x); scale bar = 200 µm and 50 µm. **E**. A part of tuber section after Lugol’s solution treatment, note numerous starch grains fill the cells; scale bar = 100 µm. (**F**). SEM, of the outer part of the tuber, note differences in sizes between starch grains in parenchyma cells (arrows, Pc) and epidermal cells (star, EC); scale 50 µm.

**Figure 4:**
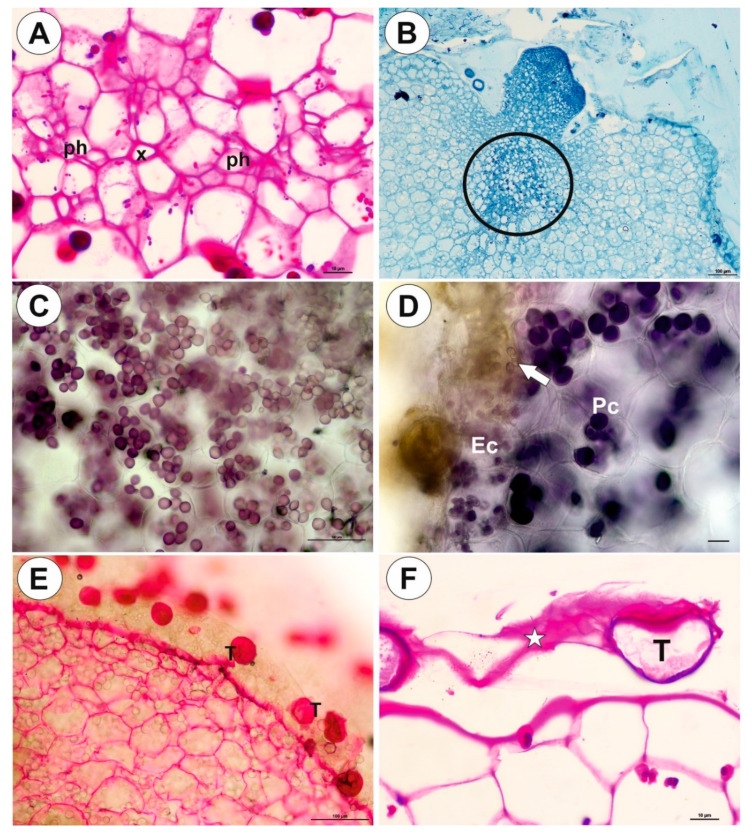
Histochemistry of *Genlisea tuberosa* tubers. (**A**). Vascular bundle, PAS reaction, note small starch grains: phloem (ph), xylem vessel (x); scale bar = 10 µm. (**B**). Tuber section stained with naphthol blue black for protein, note accumulation of protein storage vacuoles accumulation in cells (circle) near bud; scale bar = 100 µm. (**C**). Simple starch grains in parenchyma cells, Lugol’s solution treatment; scale bar = 100 µm. (**D**). Starch grains in parenchyma cells (Pc) and composed starch grains (arrow) in epidermal cells (Ec); scale bar = 20 µm. (**E**). Tuber section treated with ruthenium red: note positive reaction in cell walls of parenchyma cells and head cells of glandular trichomes (T)); scale bar = 100 µm. (**F**). Positive result of the PAS reaction of material (star) secreted by trichomes (T); scale bar = 10 µm.

Up to three tubers occur per plant. The tubers were white-yellow in color, ovoid-shaped (Figure 1A–C). The tuber is attached to the plant by a stalk (Figure 2A). At the tuber pole, where there was stalk, the developing primordia of new organs were observed in the bud (Figure 2B). The tuber surface was covered by mucilage, debris and fungal hyphae (Figure 2C,D). Small epidermal trichomes occurred, each consisting of one basal cell, one short, pedestal cell and a head cell (Figure 2C–E). The lateral wall of the pedestal cell was impregnated by cutin (Figure 2E). The head cell was globular shaped. These trichomes produced mucilage. Stomata were present on the tuber surface (Figure 2C,F). Both open and closed stomata were observed (Figure 2F). As shown in the transverse sections, the tuber was round (Figure 3A,B). The parenchymatous cortex was well developed. Vascular bundles (about 10) formed a ring, with centrally located additional vascular bundles (Figure 3B–D) that showed evidence of branching within the tuber. Within these vascular bundles, there were two groups of phloem cells near the xylem. The xylem is composed of one or two tracheary elements with evident vessels. The vasculature associated with the central part of pith comprised a vessel surrounded by radially elongated parenchymatous cells (Figure 3D). In the pith, there were intercellular spaces. Parenchyma cells of both cortex and pith were highly vacuolated and rich in large starch grains (SGs) (Figure 3E). Smaller SGs also occurred in the epidermal cells (Figure 3F, Table 1). Small SGs were in parenchyma cells of vascular bundles (Figure 4A). SGs, in parenchyma cells of both cortex and pith, were simple (Figure 4C), but in epidermal cells, mixed configurations comprising compound and simple grains co-occurred in the same cell (Figure 4D). Protein storage vacuoles were recorded in the pith near the place where the new bud was formed (Figure 4B). Ruthenium red stained cell walls of parenchyma (Figure 4E), but also head cells of trichomes (Figure 2D and Figure 4E), indicating pectins and mucilage. The PAS (periodic acid-Schiff) reaction revealed that material secreted by trichomes was most likely a polysaccharide (Figure 4F).

### 2.2. Utricularia menziesii R.Br.

**Figure 5:**
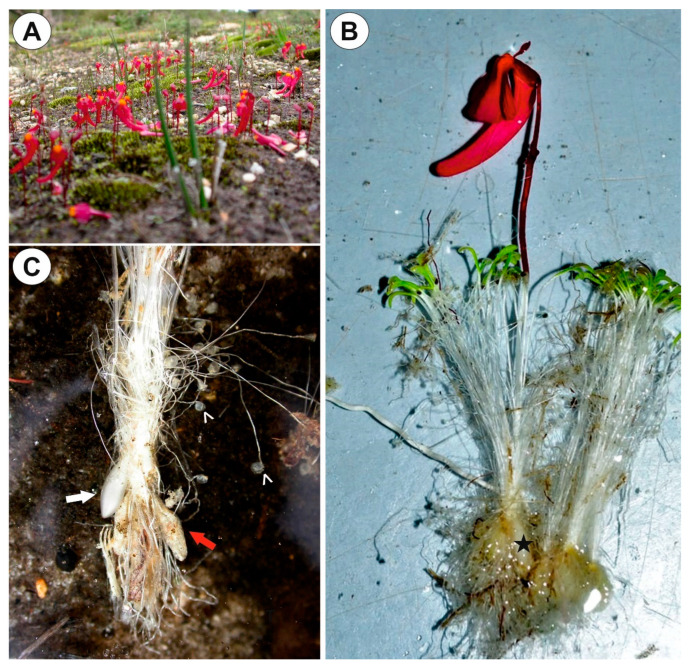
*Utricularia menziesii* in natural habitat, the Alison Baird Reserve (Yule Brook) in Western Australia. (**A**). Habitat of *Utricularia menziesii,* (photo Hans Lambers). (**B**). Excavated plants; tuber (star), author of photo André J. Arruda. (**C**). A part of excavated plant with traps (arrowhead) and tubers: young tuber (white arrow), old tuber (red arrows) (photo Hans Lambers).

**Figure 6:**
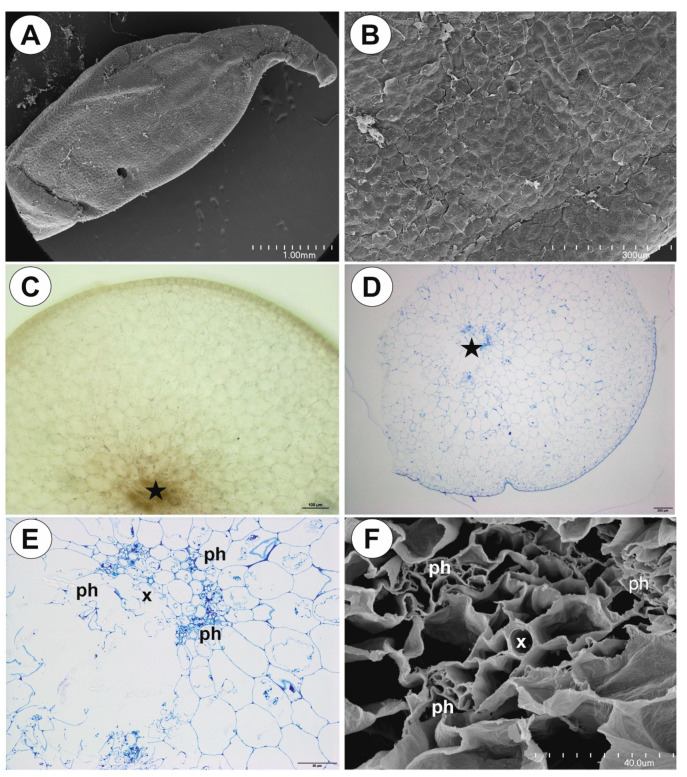
Tuber structure of *Utricularia menziesii*. (**A**). SEM, morphology of tuber; scale bar = 1mm. (**B**). SEM, micromorphology of tuber surface; scale bar = 300 µm. (**C,D**). General tuber anatomy. Note the vascular tissues (black star); scale bar = 100 µm and scale bar = 200 µm. (**E,F**). Structure of vascular tissues: phloem (ph), xylem (x); (**F**). SEM; scale bar = 50 µm and scale bar = 40 µm.

**Figure 7:**
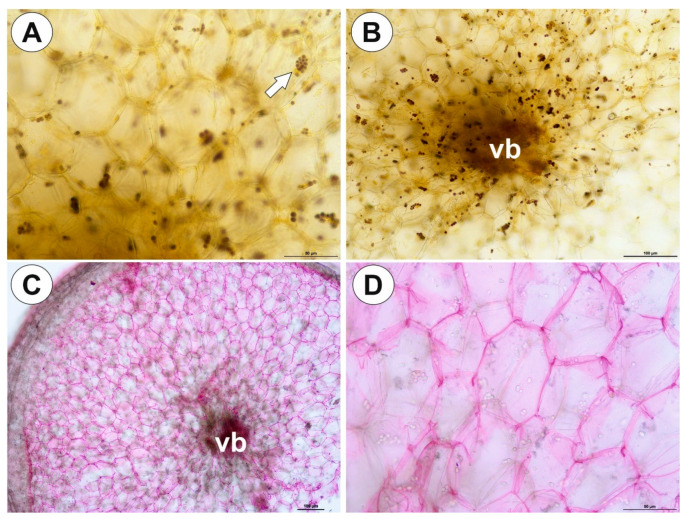
Histochemistry of *Utricularia menziesii* tubers. (**A,B**). Tuber section after Lugol’s solution treatment: compound starch grain (arrow), vascular bundle (vb); scale bar = 50 µm and scale bar = 100 µm. (**C,D**). Tuber sections treated with ruthenium red: note positive reaction in case cell walls of parenchyma cells; vascular bundle (vb); scale bar = 100 µm and scale bar = 50 µm.

The tubers were white, turgid, ovoid (Figure 5B,C) attached to a highly abbreviated stem. Old tubers (from the previous season) were flattened and comprised no obvious intact storage cells (Figure 5C and Figure 6A). The tuber was attached to the plant with a stalk, at the other tuber pole no buds or other organs were found. The tuber surface was covered by mucilage, debris and fungal hyphae, nevertheless, no trichomes were recorded (Figure 6B). As shown in the transverse sections, the tuber was round (Figure 6C,D) with a well-developed parenchymatous cortex. There was no clear border between the cortex and pith. Vascular tissues: phloem and xylem were located centrally in the organ (Figure 6C,D). Xylem with one tracheary element and a vessel element surrounded by phloem (Figure 6E,F). Although the xylem and phloem elements were distant from each other, this structure may be treated as a single vascular bundle. Parenchyma cells were highly vacuolated and contained small SGs (Figure 7A,B, Table 1). There were uniform simple and compound SGs (Figure 7A). More SGs occurred in parenchyma which surrounded the vascular tissues (Figure 7B). Mucilage in parenchyma cells was absent, and ruthenium red staining only occurred in the cell walls (Figure 7C,D).

### 2.3. Utricularia mannii Oliv. 

**Figure 8:**
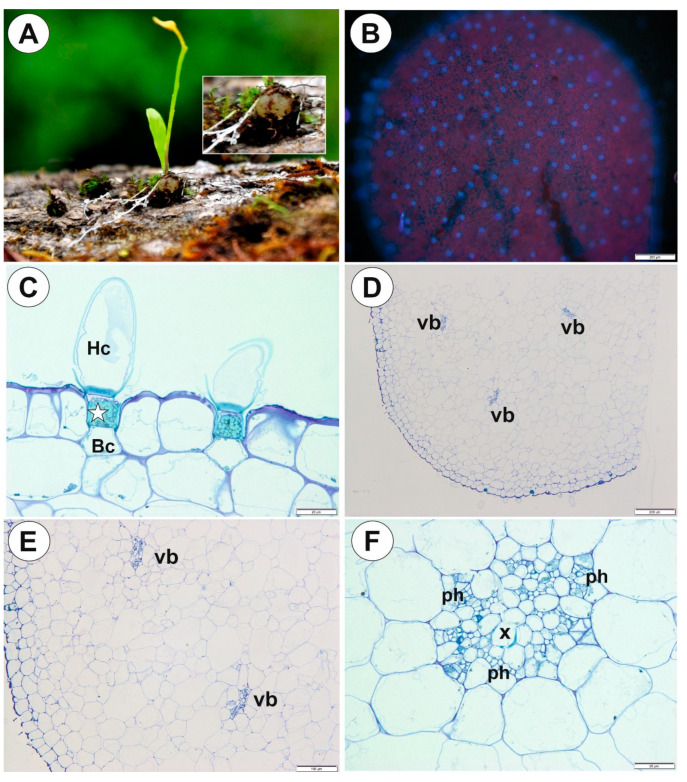
Tuber structure of *Utricularia mannii*. (**A**). *Utricularia mannii* habitat, Cameroon, Africa; (photo Dr. Ľuboš Majeský), insert – tuber. (**B**). Surface of tuber covered by numerous glandular trichomes, note bright blue fluorescence of trichome pedestal cells and red fluorescence of chlorophyll – chloroplast in epidermal cells and parenchyma cells; scale bar = 10 µm. (**C**). Structure of epidermal glandular trichome: basal cell (Bc), pedestal cell (star), head cell (Hc); scale bar = 20 µm. (**D,E**). General tuber anatomy. Note the vascular bundles (vb); scale bar = 200 µm and scale bar = 100 µm. (**F**). Structure of vascular bundle: phloem (ph), xylem vessel (x); scale bar = 20 µm.

**Figure 9:**
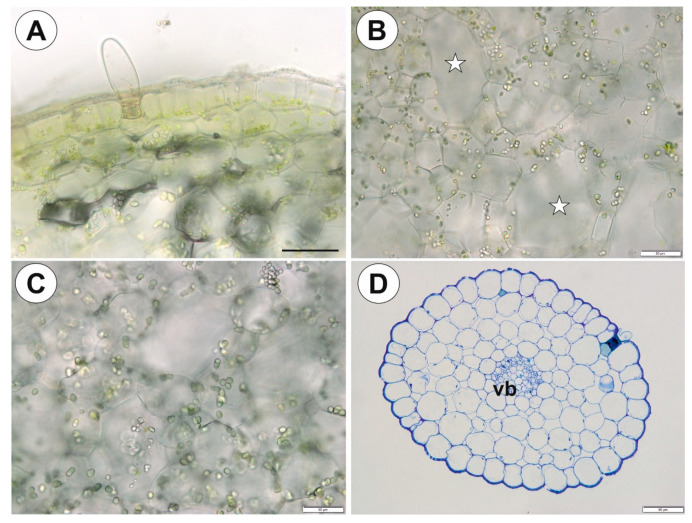
Tuber and stolon structure of *Utricularia mannii*. (**A**). Chloroplasts in epidermal cells and parenchyma; scale bar = 50 µm. (**B,C**). Starch grains in the parenchyma cells, note well developed intercellular spaces; scale bar = 50 µm. (**D**). Stolon anatomy, vascular bundle (vb); scale bar = 50 µm.

The tubers were green in color, obovoid to globose (Figure 8A). Epidermal trichomes were numerous (Figure 8B), each consisting of one basal cell, one short, pedestal cell and a head cell (Figure 8C). The lateral wall of the pedestal cell was impregnated by cutin (Figure 8B,C). The head cell was strongly elongated (Figure 8C). As shown in the transverse sections, the epidermis and parenchymatous cortex surrounded a large central cylinder (Figure 8D). Vascular bundles formed a ring, with centrally located additional vascular bundles that were larger than the perimeter bundles (Figure 8E,F). Xylem elements were surrounded by phloem (Figure 8F). Chloroplasts occurred in epidermal cells and parenchyma (Figure 9A). Intercellular spaces were well developed and were present both in the cortex and pith (Figure 9B). Parenchyma cells were highly vacuolated and contained small SGs (Figure 9C, Table 1). There were uniform simple SGs and also compound. The stolon had a similar vascular anatomy to the tuber (Figure 9D) though restricted to just one vascular bundle (Figure 9D).

### 2.4. Phylogenetic Analyses

The concatenated matrix comprehends an alignment of 212 DNA sequences of different species of Lentibulariaceae: 20 of *Genlisea*, 126 of *Utricularia*, and 66 sequences of *Pinguicula* used as the outgroup. The analyses were performed in 4012 characters of which 1755 were parsimony-informative.

**Figure 10:**
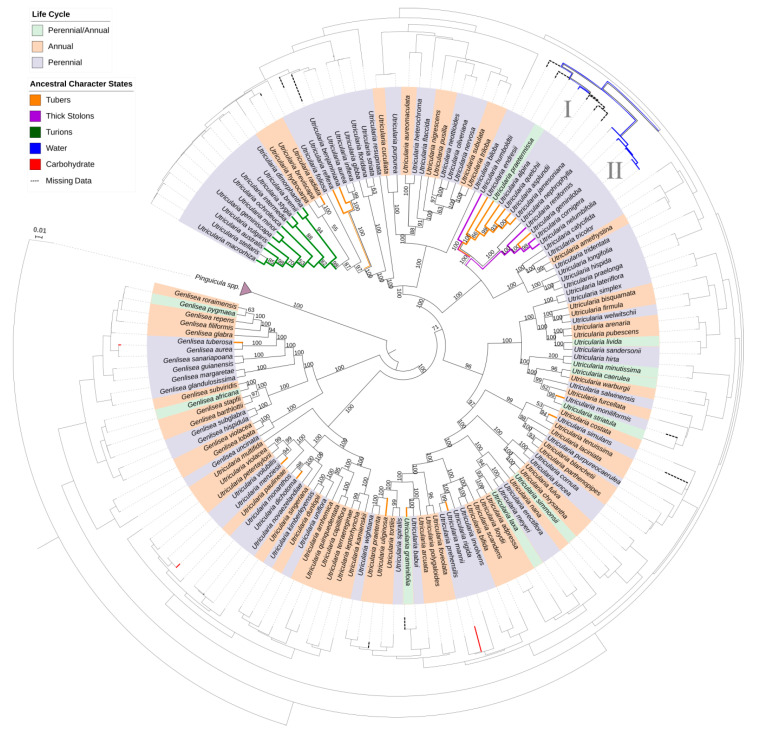
Phylogenetic trees were reconstructed with the Maximum Likelihood method. The inner tree and outer tree indicate the ancestral character states reconstructed according to parsimony criteria. The inner tree shows the possible ancestral character states of tubers, thick stolons and turions (orange, purple and green branch color, respectively). Branch numbers correspond to ultrafast bootstrap values. The outer tree shows the possible ancestral character states of the tuber and thick stolons content: carbohydrate in red; water in blue; dashed lines correspond to unknown data. The names of species are colored according to life cycle: purple for perennial, orange for annual and green for annual and/or perennial species according to Taylor [14] and Fleischmann [34]. I and II denote potential points of tuber appearance. *Pinguicula* species were used as the outgroup.

The ancestral character tracing (Figure 10, inner tree) shows that stolons have changed to tubers once in *Genlisea* and that tubers are highly homoplasious in *Utricularia*, and occurred at least 11 times in the *U. radiata*, *U. reflexa*, *U. benjaminiana*, sect. *Orchidioides*, *U. moniliformis*, *U. simulans*, *U. mannii*, *U. graminifolia*, *U. uliginosa*, *U. dichotoma* and *U. menziesii* groups. However, for the *U. reflexa*-*U. benjaminiana*-*U.aurea*-*U. inflexa* clade, tubers possibly emerged from an ancestor with a stoloniferous habit or that was already bearing tubers. But, for sect. *Orchidioides*, the two most parsimonious hypotheses for tubers appearance are: (1) that thick stolons and tubers originated from simple (thin) stolons (Figure 10, as indicated by I: thin stolon -> thick stolon [one step] or thick stolon -> tuber [one step]), or (2) that thick stolons originated from simple (thin) stolons with the thickened stolons originating the tubers (Figure 10, indicated by II, in two steps: thin stolon -> thick stolon -> tuber). 

Tubers with carbohydrate content evolved at least five times independently in the Lentibulariaceae: in *Genlisea tuberosa*, *Utricularia menziesii*, *U. mannii* lineages (Figure 10, outer tree), *U. brachiata* and *U. inflata*. In addition, water storage is found as a synapomorphy, thus only derived once with thick stolons and tubers in sect. *Orchidioides*. 

In addition, ancestral character tracing (Figure 10) shows that turions have appeared as a novel adaptation at least once in *Utricularia*, within the species of sect *Utricularia* from temperate climates.

## 3. Discussion

Darwin [30] examined tubers of *Utricularia alpina* (as *Utricularia montana*) and concluded that this species accumulates water that enable the plant to survive seasonal drought. This was later confirmed by Adlassnig [35] and Rodrigues et al. [21]. In addition, other species from sect. *Orchidioides* produce water-storing tubers or thick stolons [21]. Compton [36] studied tubers of *U. brachiata* (sect. *Phyllaria*) and found tuber parenchyma contained starch grains (SG). He concluded the water-storing function is a secondary with the primary function being carbohydrate storage. We found that *Genlisea tuberosa*, *Utricularia menziesii* and *U. mannii* form carbohydrate-rich tubers. SGs exhibited different morphologies and sizes depending on the species. The largest SGs occurred in parenchyma cells of *Genlisea tuberosa* tubers. The highest number of SGs was recorded in *Utricularia menziesii*. Accumulation of SGs was recorded also in other Lentibulariaceae perennating organs such as *Pinguicula* hibernacula ([9]; Płachno unp.) and *Utricularia* turions (e.g., [19]). Carbohydrate-rich tubers were recorded in the Lentibulariaceae in *Genlisea tuberosa* and four species of *Utricularia* that are not closely related (subgen. *Polypompholyx*: sect. *Pleiochasia U. menziesii*, subgen. *Bivalvaria*: sect. *Phyllaria U. brachiata,* sect. *Chelidon U. mannii* and subgenus *Utricularia*: sect. *Utricularia U. inflata*). Thus, these starch-rich tubers evolved at least five times independently in the Lentibulariaceae lineages. Nonetheless, further investigations are required across storage structures in the family, for example in another two species in sect. *Phyllaria* showing 1–2-mm-thick tubers, probably also serving as starch-rich vegetative propagules as found in *U. christopheri* and *U. forrestii* [14].

Regardless of whether the tubers accumulate starch grains or water, the tubers have well-developed parenchyma, which perform a storage function. Epidermal trichomes produce mucilage that may additionally protect the tuber surface. Mucilage may also interact with microflora and fungi though the function of such co-associating microorganisms is unclear. 

Tubers exhibit different vascularization; however, in all species, phloem and xylem elements are evident. The morphological and anatomical tuber characters enable these species to occupy otherwise hostile ecological niches through perennation structures that survive periods of drought. However, tuber vascularization is most likely more related to evolutionary history of species than to organ specialization.

In seasonally dry environments such as the Brazilian *Cerrado* and *kwongkan* of south-western Australia the ability to sequester nutrients, moisture and energy in underground organs represents a key strategy for survival [37]. Carbohydrates are stored in various types of organs: tubers, corms, bulbs, rhizomes, rhizophores, tuberous roots, lignotubers, and xylopodia. Starch is the most abundant storage material, with combinations of insoluble and soluble carbohydrates occurring in underground storage organs [37,38,39]. 

In *Genlisea tuberosa* and *Utricularia menziesii*, the subterranean tubers act as a key structure and bud repository able to withstand seasonal drought, summer temperatures and the periodic passage of fire (soil is good heat insulator; see [40]). Several fire adaptations are found in endemic *Cerrado* flora, including functionally herbaceous or woody geoxylic suffrutices (the ‘underground trees’, see [41]) with enlarged underground xylopodia, lignotubers, thick corky bark, thick shoots, leaves congested at shoot tips and also specialized flowering and fruiting phenologies [42]. However, there is little known of the morphological and anatomical bases to ecological adaptation in tuberous structures (see [43]). 

*Utricularia menziesii* has adopted a growth and development phenology that is similar to that found in most other herbaceous perennial geophytes that occur in the same habitat [22]. Dry season (summer) dormancy occurs from November to May, with sprouting occurring in concert with the onset of cooler and wetter winter conditions. Within a month of sprouting, tuber primordia are evident and these extend and enlarge over the growing season as the parent tubers shrivel and wither. The tuber lacks protective oversummering structures found in many other herbaceous perennial geophytes in the area where *U. menziesii* grows. For example, common geophytic *Drosera* accumulate around the tuber paper-like tissues from spent tubers that act to enclose the tuber and function to enhance water retention [22]. Though *Utricularia menziesii* grows in wet sandy swamps to moss aprons on granite rocks it is surprising that no other *Utricularia* in the southwest Australian region, a noted hotspot for carnivorous plants, have developed any form of vegetative perennation. 

Tubers from sect. *Orchidioides* and secondary tubers of *Utricularia mannii* are tuberized (enlarged) stolons [14,21,44], which can continue apical growth to form the tuberous structures. In *U. alpina,* tubers may have also lateral branches—stolons bearing traps (see illustration in Darwin [30]). In contrast to that, tubers of *Genlisea tuberosa* and *U. menziesii* have reduced apical growth and no lateral additional organs. According to Taylor [14], *U. mannii* grows as an epiphyte on mossy tree trunks; however, this species may grow on rocks as a lithophyte (Figure 8A; Ľuboš Majeský personal observations). Thus, tubers of this species may also have a water-storing function. In addition, Compton [36] proposed both starch and water storage for tubers of *U. brachiata*. Therefore, the division into tubers which storage only water or only carbohydrates represents an artificial separation.

However, there are some similarities and differences in tuberous structures in the Lentibulariaceae. Epidermal trichomes occur in tubers of *Genlisea tuberosa*, *U. mannii* and tubers and thick stolons of species *Utricularia* from sect. *Orchidioides* [21]. Darwin [30] did not observe intercellular spaces in tubers of *Utricularia alpine* however in this study they were found in tubers of *U. mannii*. Well-developed lacunae were observed in *Utricularia nelumbifolia* stolons [21]. In the study species, there was no clear partitioning between cortex and pith, but this border is clearly seen in tubers and thick stolons of species *Utricularia* sect. *Orchidioides* [21]. There are also clear differences in vascularization in tubers of *Genlisea tuberosa*, *Utricularia menziesii* and *U. mannii* where phloem and xylem formed vascular bundles, but in the tubers and thick stolons of species *Utricularia* from sect. *Orchidioides*, [21] the xylem and phloem elements are separated. Vascular bundles with phloem groups flanking the xylem were observed in stolons of *Utricularia dichotoma* [6]. In tubers of *Genlisea tuberosa* and *U. mannii*, vascular bundles are branching in contrast to the tuber of *Utricularia menziesii*, with single vascular bundle in the tuber. With single, centrally located vascular bundles, tubers of *Utricularia menziesii* resembles the classical anatomy of root tubers (a ring of collateral bundles is typical for stem tubers in eudicots); however, *Utricularia* species do not produce roots [14]. Compton [36] also noted single vascular bundles in tubers of *U. brachiata*. This type of tuber originates from a stolon with one vascular bundle. Such stolons are known for species from section *Pleiochasia*, to which *U. menziesii* belongs [6].

For *Utricularia mannii*, the tubers are very similar to the tubers of species *Utricularia* from sect. *Orchidioides*, particularly the occurrence of chlorophyll and glandular trichomes. However, they differ in starch and vascular tissue development.

The phylogenetic hypothesis (Figure 10) are in general congruent to previously published studies [12,27,33,45,46,47,48]. Indeed, we used accessible public databases (mostly from Westermeier [46] and Silva [45]), and performed the analyses using a supermatrix approach with two concatenated chloroplast regions. One is a phylogenetically informative marker for *Utricularia* and *Genlisea* species, the *mat*K gene with *trn*K intron, while the other is the *rbc*L gene, which is more conserved for these genera. Therefore, the polymorphic nature of the *mat*K/*trn*K region and the conservation of *rbc*L [49,50] could provide greater resolution. Considering the two main clades with perennating organs, and ignoring the situation in which these organs are autapomorphic (*Genlisea tuberosa*, *Utricularia menziesii, U. dichotoma, U. uliginosa, U. graminifolia, U. mannii, U. simulans, U. moniliformis* and *U. radiata*), thick stolons and tubers for sect. *Orchidioides* (Figure 10, I and II) and turions for sect. *Utricularia* are present in lineages with relatively short branches in comparison with other groups (see the outer tree, Figure 10), even regarding the several factors that could reflect in mutation rates, as body size, population dynamics, and lifestyle among others [51,52]. Taking into account that we applied plastidial DNA sequences, the generation time effect and life history may also affect the rates of molecular evolution in flowering plants (e.g., [53]). This could explain this pattern for lineages of sect. *Orchidioides* and sect. *Utricularia*.

## 4. Materials and Methods

Material of *Genlisea tuberosa* Rivadavia, Gonella & A.Fleischm. was collected in the Serra da Canastra region, southern Minas Gerais State (Southeastern Brazil), in campos *rupestres* (rupestrian grasslands) of the *Cerrado* (collecting permits was ICMBio/MMA/SISBIO #74307-1). *Utricularia menziesii* R.Br. was collected from the Alison Baird Reserve (Yule Brook) in Western Australia, a privately owned and managed nature reserve. *Utricularia mannii* Oliv. (from Mount Cameroon, Cameroon, Africa) was cultivated in the Department of Plant Cytology and Embryology, Jagiellonian University in Kraków. Tubers were fixed as below for anatomical and histochemical studies.

The tubers were examined using light microscopy (LM) and scanning electron microscopy (SEM) as follows. Material was fixed in a mixture of 2.5% or 5% glutaraldehyde with 2.5% formaldehyde in a 0.05 M cacodylate buffer (Sigma-Aldrich, Sigma-Aldrich LLB, Poznan, Poland; pH 7.2) overnight or for several days, washed three times in a 0.1 M sodium cacodylate buffer and post-fixed in a 1% osmium tetroxide solution at room temperature for 1.5 h. Later material was treated as previously [54]. The semi-thin sections (0.9–1.0 µm thick) prepared for the LM were stained with aqueous methylene blue/azure II (MB/AII) for 1–2 min [55] and examined using an Olympus BX60, as well as Nikon Eclipse E400 light microscope for the general histology. The periodic acid-Schiff (PAS) reaction for the LM (semi-thin sections) was also used to reveal the presence of insoluble polysaccharides.

Materials were also embedded in Technovit 7100 (Kulzer, Germany) for further histological analysis. This material was fixed (as above), washed three times in a 0.1 M sodium cacodylate buffer, dehydrated in a graded ethanol series for 15 min at each concentration and kept overnight in absolute ethanol. Later, the samples were infiltrated for 1 h each in 3:1, 1:1 and 1:3 (*v*/*v*) mixtures of absolute ethanol and Technovit and then stored for 12 h in pure Technovit. The resin was polymerised by adding a hardener. Materials were also sectioned to 5 μm thick using a rotary microtome, stained with 0.1% toluidine blue O (TBO) and mounted in DPX (Sigma-Aldrich, Sigma-Aldrich LLB, Poznan, Poland). The selected Technovit sections were stained with naphthol blue black (NBB) for total protein staining or the periodic acid-Schiff (PAS) reaction was performed for starch visualization.

In order to identify the main classes of the chemical compounds that are present in the tubers, histochemical procedures with the fixed tubers using Lugol’s solution were performed to detect the starch grains and proteins [56]. 0.1% ruthenium red was used for pectin and mucilage detection [57,58]. 

Tubers were cut using a razor blade and observed under UV light using an Olympus BX60, as well as Nikon Eclipse E400 light microscope to determine: cell walls impregnated with cutin and autofluorescence of chlorophyll. 

For the SEM, tubers were fixed (as above) and later dehydrated and critical point dried using CO_2_. They were then sputter-coated with gold and examined at an accelerating voltage of 20 kV using a Hitachi S-4700 scanning electron microscope (Hitachi, Tokyo, Japan), which is housed in the Institute of Geological Sciences, Jagiellonian University in Kraków, Poland.

We measured starch grain diameter for each species (Table 1) as follows. For each species, one randomly chosen tuber section was selected. The number of starch grain measurements per cell type of a particular species was 100. Each variable was tested using the Shapiro–Wilk W-test for normality. The homogeneity of variance was estimated with Levene’s test. Statistical differences in starch grains diameter in each species were assessed using one-way ANOVA, followed by Tukey’s post-hoc comparison test. Statistical analyses were performed on the raw data using Statistica 13 software (StatSoft Inc., Oklahoma, USA). Data from measurements of starch grain diameter were expressed in µm as mean ± SD. Data were considered statistically significant at *** *p* < 0.001. We measured also number of starch grains per 100 µm^2^ (Table 2). For each species one randomly chosen tuber section was selected.

To reconstruct the phylogenetic hypothesis, we aligned sequences of chloroplast regions *mat*K/*trn*K and *rbc*L genes from GenBank Nucleotide database (Table S1) using MAFFT version 7 [59] with default parameters and generated a supermatrix with FASConCAT-G version 1.04 [60]. With the resulting supermatrix we calculated a Maximum Likelihood phylogenetic trees with IQ-TREE version 1.6.12 [61] using TVM+F+I+G4 model parameters chosen according with AIC criterion [62] using ModelFinder [63] implemented in IQ-TREE. Clade support was evaluated using ultrafast bootstrap [64] with 1000 replicates. Gaps were treated as missing data. The ancestral state reconstruction was performed using Maximum Parsimony criteria from a character matrix developed according to data from the present study and previous publications [12,14,20,21,34] to map the absence or presence of turions, tubers, thick stolons and the type of storage material into the molecular phylogeny using Mesquite version 3.61 [65]. The final tree was edited using Interactive Tree of Life (iTol) version 5.5.1 [66].

## 5. Conclusions

In contrast to species from section *Orchidioides*, which produce tubers storing mainly water, *Genlisea tuberosa*, *Utricularia menziesii* and *U. mannii* form starch-rich tubers. Such tubers evolved independently at least five times in the family Lentibulariaceae. In *Genlisea tuberosa* and *Utricularia menziesii,* underground tubers are a perennating structure that are key strategy to survive in summer fire-prone environments. In contrast to examined species here, in tubers of species from section *Orchidioides* the xylem and phloem elements are separated from each other which supports molecular studies from previous studies that this species is not related to species from *Orchidioides*. In contrast to most *Utricularia* and *Genlisea* lineages, where tubers evolved from regular stolons, sect. *Orchidioides* probably evolved through a continuum of transformation going from stolons, thick stolons to tubers. The occurrence of stomata on the underground tubers of *G. tuberosa* is unusual and point to a stem-based origin for the tuber tissue. Both tubers of *G. tuberosa*, and *U. mannii* had epidermal trichomes, which produce mucilage for protection. In *G. tuberosa* and *U. mannii,* vascular bundles formed a ring, but there was a centrally located additional vascular bundle. However, in *U. menziesii*, the single vascular bundle was located centrally in the tuber.

## Figures and Tables

**Table 1:** Starch grain diameter in *Genlisea tuberosa*, *Utricularia mannii* and *U. menziesii*.

Species	Cell Type	Diameter of Starch Grains (mean ± SD)
*Genlisea tuberosa*	Parenchyma	17.34 ± 3.07 µm
	Epidermis	7.57 ± 1.53 µm
*Utricularia mannii*	Parenchyma	7.01 ± 1.46 µm
*Utricularia menziesii*	Parenchyma	3.76 ± 0.70 µm

**Table 2:** Number of starch grains per 100 µm^2^ in *Genlisea tuberosa*, *Utricularia mannii* and *U. menziesii.*

Species	Number of Starch Grains Per 100 µm^2^ (mean ± SD)
*Genlisea tuberosa*	22.6 ± 3.59
*Utricularia mannii*	32.0 ± 5.83
*Utricularia menziesii*	36.4 ± 5.85

## Supplementary Materials

Supplementary Materials can be found at https://www.mdpi.com/1422-0067/21/14/5143/s1. Table S1. Data used for the phylogenetic analyses. “-” denotes missing data. Pinguicula species were 20 used as outgroup.
