# Supplementary material for: Structural Features of Carnivorous Plant (Genlisea, Utricularia) Tubers as Abiotic Stress Resistance Organs
Source: Int J Mol Sci. 2020 Jul 21;21(14):5143. doi: 10.3390/ijms21145143 (PMC7403982; doi:10.3390/ijms21145143)
Supplement: Supplementary file 1 [file ijms-21-05143-s001.pdf]

International Journal of Molecular Sciences

MDPI

Article – Supplementary Materials

Structural features of carnivorous plant (*Genlisea*, *Utricularia*) tubers as abiotic stress resistance organs

Bartosz J. Płachno [1,*], Saura R. Silva [2], Piotr Świątek [3], Kingsley W. Dixon [4], Krzystof Lustofin [1], Guilherme C. Seber [2] and Vitor F. O. Miranda [2]

[1] Department of Plant Cytology and Embryology, Institute of Botany, Faculty of Biology, Jagiellonian University in Kraków, Gronostajowa 9 St. 30-387 Cracow, Poland; krzysztof.lustofin@doctoral.uj.edu.pl (K.L.)

[2] São Paulo State University (Unesp), School of Agricultural and Veterinarian Sciences, Laboratory of Plant Systematics, Jaboticabal, CEP 14884-900, SP, Brazil; saura.silva@gmail.com (S.R.S); gcseber@gmail.com (G.C.S.); vitor.miranda@unesp.br (V.F.O.M.)

[3] Faculty of Natural Sciences, Institute of Biology, Biotechnology and Environmental Protection, University of Silesia in Katowice, Jagiellońska 28, 40-032 Katowice; piotr.swiatek@us.edu.pl

[4] School of Molecular and Life Sciences, Curtin University, Kent Street, Bentley, Perth, Western Australia 6102, Australia; kingsley.dixon@curtin.edu.au

* Correspondence: bartosz.plachno@uj.edu.pl

Table S1. Data used for the phylogenetic analyses. “-” denotes missing data. *Pinguicula* species were used as outgroup.

Species	*mat*K/*trn*K	*rbc*L
Genlisea africana	FN641702	-
Genlisea aurea	NC037078	NC037078
Genlisea barthlottii	FN641704	-
Genlisea filiformis	NC037079	NC037079
Genlisea glabra	FN641692	-
Genlisea glandulosissima	FN641700	-
Genlisea guianensis	FN641696	AY128631
Genlisea hispidula	FN641705	-
Genlisea lobata	FN641711	-
Genlisea margaretae	HG530134	HG530134
Genlisea pygmaea	NC037080	NC037080
Genlisea repens	NC037081	NC037081
Genlisea roraimensis	AF531817	-
Genlisea sanariapoana	FN641698	-

Genlisea stapfii	AF531818	-
Genlisea subglabra	FN641706	-
Genlisea subviridis	FN641703	-
Genlisea tuberosa	NC037082	NC037082
Genlisea uncinata	AF531819	-
Genlisea violacea	NC037083	NC037083
Utricularia adpressa	MF765548	AF482527
Utricularia alpina	AF531822	AF482528
Utricularia amethystina	MN223721	MN223721
Utricularia arcuata	MF765518	-
Utricularia arenaria	MF765500	MF991480
Utricularia arnhemica	MF765494	
Utricularia asplundii	MF765551	-
Utricularia aurea	KX604176	MF991481
Utricularia aureomaculata	MF765532	-
Utricularia australis	AF531823	MF572257
Utricularia babui	MF765516	-
Utricularia benjaminiana	MF765539	-
Utricularia bifida	MF765522	MF991483
Utricularia biloba	MF765534	-
Utricularia bisquamata	MF765503	-
Utricularia blanchetii	AF531841	-
Utricularia bremii	MF765536	MF991484
Utricularia breviscapa	MF765537	MF991487
Utricularia caerulea	MF765507	MH292762
Utricularia calycifida	AF531824	MF991489
Utricularia capilliflora	MF765495	-
Utricularia chrysantha	MF765527	-
Utricularia cornigera	KY689699	-
Utricularia cornuta	MF765524	MK526851
Utricularia costata	-	AY128628
Utricularia cucullata	-	MF991493
Utricularia dichotoma	AF531826	MK959214
Utricularia dimorphantha	KX604186	MF991495
Utricularia dunlopii	MF765493	-
Utricularia endresii	MF765543	-
Utricularia erectiflora	MF765514	-
Utricularia firmula	MF765501	-
Utricularia flaccida	AF531830	-
Utricularia floridana	MF765541	KY627462
Utricularia foliosa	MF765538	MF991499

Utricularia foveolata	AF531850	-
Utricularia fulva	MF765526	-
Utricularia furcellata	KY490353	-
Utricularia geminiloba	KX604216	-
Utricularia geminiscapa	-	MG223028
Utricularia gibba	KC997777	KC997777
Utricularia graminifolia	MH292757	MH292763
Utricularia heterochroma	MF765533	-
Utricularia hirta	MF765509	MH292769
Utricularia hispida	AF531829	MF991504
Utricularia humboldtii	AF531836	-
Utricularia hydrocarpa	KX604190	MF991508
Utricularia inflexa	-	MF991509
Utricularia intermedia	AF531839	MG222643
Utricularia involvens	MF765550	-
Utricularia jamesoniana	MF765547	-
Utricularia juncea	AF531832	AY128630
Utricularia kamienskii	MF765499	-
Utricularia kimberleyensis	MF765492	-
Utricularia laciniata	KX604228	MF991513
Utricularia lateriflora	MF765504	MF991514
Utricularia laxa	MF765521	MF991568
Utricularia leptorhyncha	MF765498	-
Utricularia livida	AF531833	MF991569
Utricularia lloydii	MF765549	-
Utricularia longifolia	AF531834	MF991515
Utricularia macrorhiza	H803177	MF991517
Utricularia mannii	MF765520	-
Utricularia menziesii	-	MF991573
Utricularia meyeri	KX604231	MF991570
Utricularia minor	MF765554	MF572258
Utricularia minutissima	MF765510	MH292770
Utricularia monanthos	-	MF991519
Utricularia moniliformis	MF765508	-
Utricularia multifida	AF531848	MF991571
Utricularia nana	AF531837	MF991520
Utricularia nelumbifolia	MF765544	MF991521
Utricularia neottioides	KX604195	MF991522
Utricularia nephrophylla	AF531827	-
Utricularia nervosa	MF765552	MF991525
Utricularia nigrescens	MF765529	-

Utricularia novaezelandiae	MF765490	-
Utricularia ochroleuca	-	MF991528
Utricularia oliveriana	MF765530	-
Utricularia parthenopipes	AF531842	-
Utricularia paulineae	MF765488	-
Utricularia petertaylorii	MF765485	-
Utricularia polygaloides	MH292760	MH292764
Utricularia praelonga	AF531843	MF991572
Utricularia praeterita	MH292761	MG966528
Utricularia praetermissa	KY689698	-
Utricularia prehensilis	MF765519	-
Utricularia pubescens	AF531844	AY128629
Utricularia purpurea	AF531845	MG222116
Utricularia purpureocaerulea	MF765511	-
Utricularia pusilla	MF765531	MF991532
Utricularia quelchii	AF531846	-
Utricularia quinquedentata	MF765496	-
Utricularia radiata	KX604185	KX397996
Utricularia reflexa	MF765540	MF991534
Utricularia reniformis	KT336489	KT336489
Utricularia resupinata	MF765542	MK526854
Utricularia rigida	AF531838	-
Utricularia salwinensis	-	MF786622
Utricularia sandersonii	AF531847	MF991542
Utricularia scandens	MF765523	MH292768
Utricularia simmonsii	MF765525	-
Utricularia simplex	MF765505	-
Utricularia simulans	MF765513	MF991543
Utricularia singeriana	MF765489	-
Utricularia spiralis	AF531851	-
Utricularia stellaris	KX604182	-
Utricularia striata	-	MF991546
Utricularia striatula	KY490354	MF786551
Utricularia stygia	MF765535	MF991547
Utricularia subulata	AF531821	KY627581
Utricularia tenuissima	MF765512	-
Utricularia terraereginae	MF765497	-
Utricularia tortilis	MF765515	-
Utricularia tricolor	KX604210	MF991554
Utricularia tridentata	AF531825	MF991566
Utricularia triloba	MF765528	AF482530

Utricularia uliginosa	AF531849	MH292767
Utricularia uniflora	MF765491	MF991563
Utricularia violacea	MF765486	-
Utricularia volubilis	KX604227	MF991564
Utricularia vulgaris	AF531831	MG224009
Utricularia warburgii	MF765506	-
Utricularia welwitschii	MF765502	-
Utricularia wightiana	MH292773	MH292765
Pinguicula acuminata	DQ010652	-
Pinguicula agnata	AF531782	AY128627
Pinguicula albida	LC348432	-
Pinguicula alpina	AF531783	-
Pinguicula antarctica	DQ010653	-
Pinguicula benedicta	LC348433	-
Pinguicula bissei	LC348434	-
Pinguicula bohemica	LC348435	-
Pinguicula calyptrata	FM200225	-
Pinguicula colimensis	LC348436	-
Pinguicula conzattii	LC348437	-
Pinguicula corsica	AF531784	-
Pinguicula crassifolia	LC348438	-
Pinguicula crystallina	DQ010654	-
Pinguicula cubensis	LC348439	-
Pinguicula cyclosecta	LC348440	-
Pinguicula debbertiana	LC348441	-
Pinguicula dertosensis	LC348442	-
Pinguicula ehlersiae	HG803178	AF482523
Pinguicula elongata	FM200224	-
Pinguicula emarginata	AF531785	-
Pinguicula esseriana	DQ010656	-
Pinguicula filifolia	AF531786	MF991475
Pinguicula fiorii	AF531787	-
Pinguicula fontiqueriana	AF531788	-
Pinguicula gigantea	AF531789	-
Pinguicula gracilis	AF531790	AF482524
Pinguicula grandiflora	AF531791	AF482525
Pinguicula gypsicola	LC348444	AF482526
Pinguicula hemiepiphytica	LC348445	-
Pinguicula ibarrae	LC348446	-
Pinguicula immaculata	LC348447	-
Pinguicula involuta	FM200226	-

Pinguicula ionantha	DQ010658	-
Pinguicula jackii	LC348449	-
Pinguicula jaumavensis	LC348450	-
Pinguicula kondoi	LC348451	-
Pinguicula laueana	DQ010659	-
Pinguicula leptoceras	AF531792	-
Pinguicula lilacina	LC348452	-
Pinguicula longifolia	DQ010660	-
Pinguicula lusitanica	DQ010661	-
Pinguicula lutea	DQ010662	-
Pinguicula macroceras	AF531796	-
Pinguicula macrophylla	LC348453	-
Pinguicula medusina	LC348454	-
Pinguicula mirandae	LC348455	-
Pinguicula moctezumae	AF531797	-
Pinguicula moranensis	DQ010657	HQ384871
Pinguicula mundi	AF531800	-
Pinguicula nevadensis	DQ010664	-
Pinguicula nivalis	LC348456	-
Pinguicula oblongiloba	LC348457	-
Pinguicula planifolia	LC348458	-
Pinguicula poldinii	AF531804	-
Pinguicula potosiensis	LC348459	-
Pinguicula pumila	LC348460	-
Pinguicula ramosa	DQ010667	-
Pinguicula rectifolia	AF531801	-
Pinguicula rotundiflora	AF531802	-
Pinguicula sharpii	AF531803	-
Pinguicula vallisneriifolia	AF531805	-
Pinguicula variegata	DQ010668	-
Pinguicula villosa	DQ010669	JN965726
Pinguicula vulgaris	AF531806	MK526348
Pinguicula zecheri	LC348461	-

© 2020 by the authors. Submitted for possible open access publication under the terms and conditions of the Creative Commons Attribution (CC BY) license (http://creativecommons.org/licenses/by/4.0/).
